# 876. Renal Function, Lipid Profile, and Cardiovascular Events After Switching to Abacavir Containing Regimen in Antiretroviral-Therapy-Experienced People Living with HIV in Northern Thailand

**DOI:** 10.1093/ofid/ofab466.1071

**Published:** 2021-12-04

**Authors:** Pitchaporn Phudphong, Quanhathai Kaewpoowat, Vuddhidej Ophascharoensuk, Saowaluck Yasri

**Affiliations:** 1 Department of Internal medicine, Faculty of medicine, Chiang Mai university, Thailand, muang, Chiang Mai, Thailand; 2 Faculty of Medicine, Chiang Mai University, Thailand, Iowa City, Iowa; 3 Department of internal medicine, faculty of medicine, Chiang Mai university, Thailand, Muang, Chiang Mai, Thailand

## Abstract

**Background:**

Abacavir (ABC) is commonly used as part of antiretroviral therapy (ART) regimen for people living with HIV (PLWH) with renal dysfunction in resource limiting countries. While the renal function changes and association with cardiovascular (CV) events have been well described in developed countries, these information is limited in Asian population. Herein, this study aims to describe the changes in renal function, lipid profile and CV events after ABC switching in ART-experienced PLWH in Northern Thailand.

**Methods:**

This retrospective chart-review study was conducted among adults ART-experienced PLWH (≥18 years old) who received ABC-containing regimen during January 2016 to December 2018 at Maharaj Nakorn Chiang Mai Hospital. Demographic data, HIV-related treatments, creatinine, lipid profile and CV events were collected. Patients were categorized into early switching group and late switching group (CrCl≥50 ml/min and CrCl< 50 ml/min before switching to ABC). The change of CrCl, urinalysis profiles, lipid profiles, CD4, viral load, and cardiovascular events at 12 months after ABC initiation were assessed.

**Results:**

Total of 115 participants were enrolled with mean age of 55.2±10.7 years and 63.5% were male. Of those, 87.8% of patients had received Tenofovir disoproxil fumarate (TDF) prior to ABC. Mean of CrCl at baseline was 47.6±16.8 ml/min and at 12th month was 49.56±19.42 ml/min with mean difference of 3.7 ml/min (95%CI 1.6-5.8, P< 0.001). The improvement of CrCl at 12 months in early switching group was statistically significant compared to late switching. Other two associated factors with improved CrCl after switching to ABC were duration of TDF exposure during CrCl< 60 ml/min (OR 9.26, P 0.004) and history of protease inhibitors (PIs) exposure (OR 0.06, P 0.03). No significant changed in lipid profile, CD4 and virological outcome overtime. There were only 2 CV events observed (9.3:1000 person-year, 95%CI 2.3-37.1).

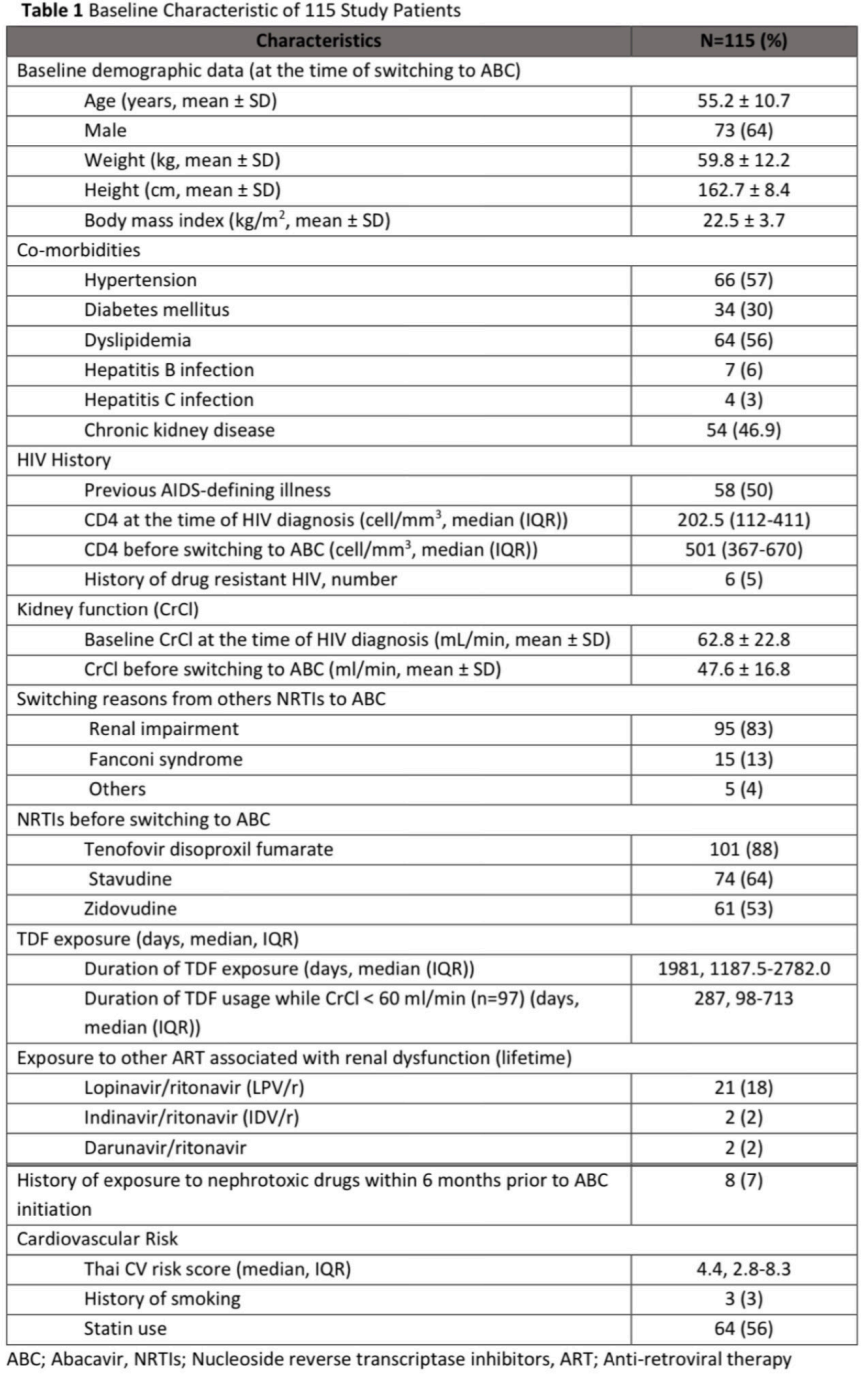

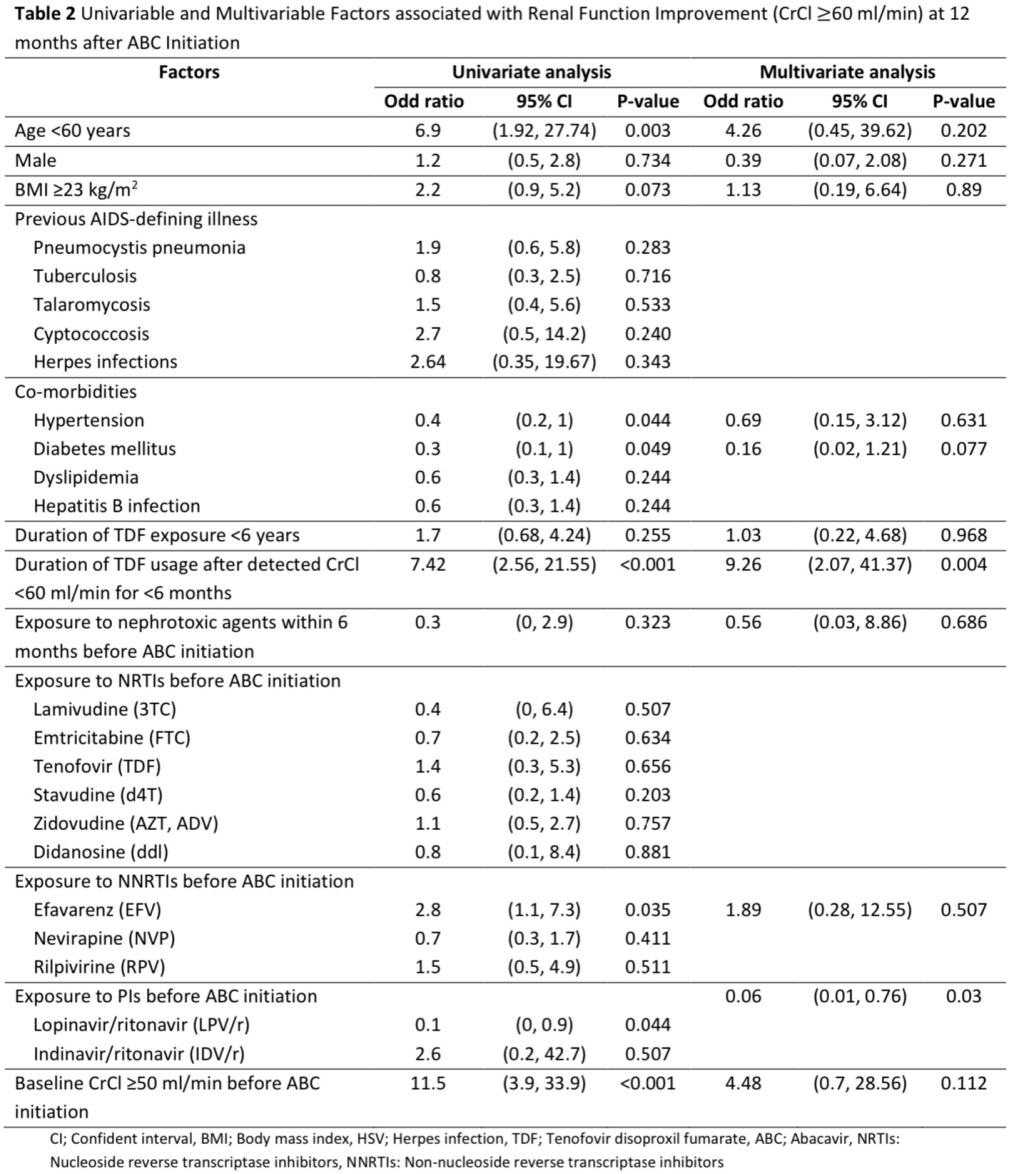

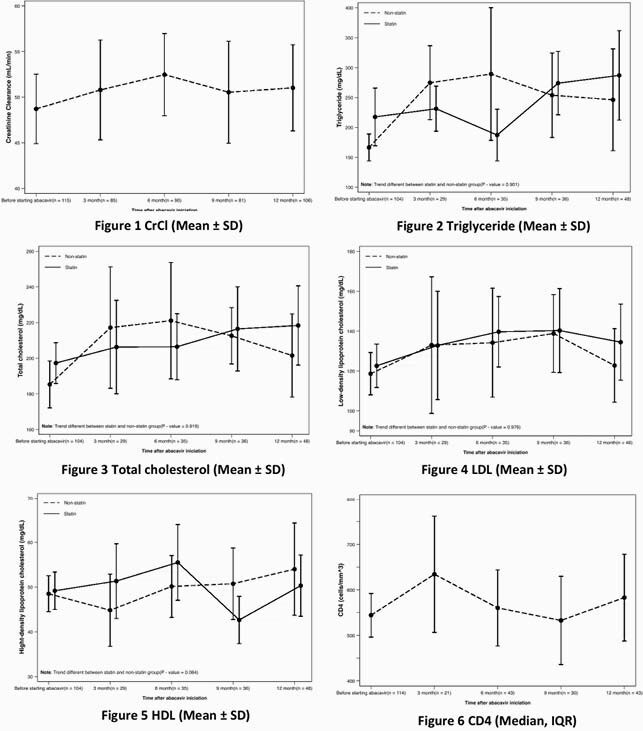

Figure. (1) Creatinine clearance (ml/min) during follow up period. (2) Triglyceride (mg/dl) during follow-up period. (3) Total cholesterol (mg/dl) during follow up period. (4) LDL (mg/dl) during follow up period. (5) HDL (mg/dl) during follow up period. (6) CD4 (cells/mm^3^) during follow up period

**Conclusion:**

ABC used in Thai ART-experienced PLWH appeared to be effective with low CV event in the first year. Despite the statistically significant in the change of CrCl after ABC switching, the change was subtle and need further evaluation.

**Disclosures:**

**All Authors**: No reported disclosures

